# Salinity stress amelioration through selenium and zinc oxide nanoparticles in rice

**DOI:** 10.1038/s41598-025-12106-3

**Published:** 2025-07-29

**Authors:** Monalisha Mishra, Shadma Afzal, Ranjana Yadav, Nand K. Singh, Saeedeh Zarbakhsh

**Affiliations:** 1 Department of Biotechnology, Motilal Nehru National Institute of Technology Allahabad, Prayagraj, 211004 India; 2 Laboratory of Bioclimatology, Department of Ecology and Environmental Protection, Poznan University of Life Sciences, Poznań, Poland 60-649; 3 Department of Horticultural Science, College of Agriculture, Shiraz University, Shiraz, Iran

**Keywords:** Antioxidant defense, Green synthesis, Nutrient uptake, Oxidative stress, Osmoprotectants, Plant physiology, Plant sciences

## Abstract

Salinity is one of the most dominant abiotic stresses limiting growth and productivity in rice (*Oryza sativa* L.), thereby posing a serious threat to global food security. To enhance plants’ tolerance to salinity stress, the application of green-synthesized nanoparticles presents a novel and eco-friendly approach. This research article investigates the ameliorative effects of selenium (Se-NPs) and zinc oxide (ZnO-NPs) nanoparticles, both individually and in combination, on rice plants under salinity stress. Our results revealed that salinity stress significantly impaired rice growth and productivity, reducing plant height, root length, and yield-related traits, including tiller count, number of grains per spike, and grain weight. Furthermore, it induced oxidative stress, as evidenced by elevated levels of malondialdehyde and proline. The elevated levels of reactive oxygen species were visibly confirmed through histochemical staining. However, treatment with Se-NPs and ZnO-NPs significantly alleviated these adverse effects by enhancing the plant’s antioxidant defense mechanism. Activities of key antioxidant enzymes such as superoxide dismutase (50.06%), catalase (59.92%), ascorbate (104.28%), and peroxidase (85%) were significantly elevated, contributing to efficient ROS scavenging and reduced lipid peroxidation. The combined nanoparticle application was particularly effective in restoring physiological and biochemical parameters to near-normal levels, with increases of 46.32% in plant height, 70.53% in root length, and 100.7% in grains per spike under salinity stress. Furthermore, the enhanced accumulation of minerals such as Zn (31.8 ppm), Se (0.57 ppm), and Fe (7.4 ppm) in rice grains was also observed, indicating a dual benefit of stress alleviation and nutritional enrichment. Green-synthesized Se-NPs and ZnO-NPs, particularly when combined, offer a promising strategy for mitigating salinity stress in rice. Beyond enhancing stress tolerance and growth, the nanoparticles also contribute to the biofortification of rice grains, thereby improving both crop resilience and nutritional value in saline environments.

## Background

Rice (*Oryza sativa* L.) is regarded as one of the first crops to be grown worldwide and is of considerable economic importance^1^. In subtropical, continental, and temperate climates, approximately 49 million hectares of land are devoted to rice cultivation^2^. The rice-wheat system (RWS) covers approximately 9.2 million hectares (mha) in India, with over 85% of that area located in the Indo-Gangetic Plains of the states of Uttar Pradesh, Punjab, Haryana, Bihar, and West Bengal^3^. It is a fundamental staple food that is consumed by about 3.5 billion people worldwide, mainly in Asia. For global food security, rice is a vital dietary energy source. However, several abiotic stresses, including heat, drought, salinity, and submersion, are threatening rice production, and their effects are expected to worsen due to climate change^4^. Furthermore, rising sea levels spurred on by climate change cause more floods and salinization and/or alkalization of agricultural land, especially along the coast. It has been estimated that by 2050, the percentage of places affected by salt will have increased by almost 50%^5^. Alkaline and/or neutral salts, including sodium carbonate (Na_2_CO_3_), sodium bicarbonate (NaHCO_3_), sodium chloride (NaCl), and sodium sulfate (Na_2_SO_4_), among others, present a significant worldwide challenge to plant growth, development, and yield in agricultural soils^6^. A quarter to 30% (about 70 mha) of irrigated land is susceptible to salinity damage, which either depletes or substantially decreases its economic production^5^.

According to several recent research studies, nanoparticles (NPs) are among the most effective substitutes for currently employed dubious methods of addressing abiotic stressors in plants. Nanotechnology, referred to as the science of the miniature, is a multidisciplinary field that combines engineering and science to develop materials at the nanoscale (nm) level, with dimensions ranging from 1 to 100 nm. These dimensions are roughly on the scale of atoms, molecules, and their macromolecular structures^7^. Given the challenges posed by climate change, environmental pollution, and world food insecurity, research into nanotechnology and its use in agronomy can be a crucial alternative for achieving sustainable agriculture. Previous research has clearly demonstrated that NPs have a positive impact on plant growth under salt stress by reducing the accumulation of excess Na^+^ in plant tissues and mitigating salinity stress. In abiotic stress conditions, NPs also enhanced osmolyte accumulation and antioxidant activity^8^. Given the increasing focus on efficient nutrient delivery systems, a variety of nano-fertilizers are being developed to improve plant growth, especially under stressful conditions. These smart nano-fertilizers, with their small particle size, high surface area-to-volume ratio, controlled release, and enhanced absorption, offer a promising alternative to traditional chemical fertilizers^9^. They not only improve nutrient efficiency and reduce production cost but also minimize the environmental impact of conventional fertilizers^10^.

Recently, smart nano-fertilizers have been developed to improve plant performance under abiotic stress. Zinc-based nanoparticles contribute to plant growth enhancement, improved crop productivity, and increased tolerance to various abiotic stressors^11^. Additionally, their efficiency in crop protection and ability to function as smart carriers for precise and controlled delivery of agrochemicals highlight their promising applications in sustainable agriculture^12^. Similarly, studies have shown that selenium nanoparticles as a promising strategy enhance plant tolerance to salinity and other abiotic stresses^13^.

**Fig. 1:**
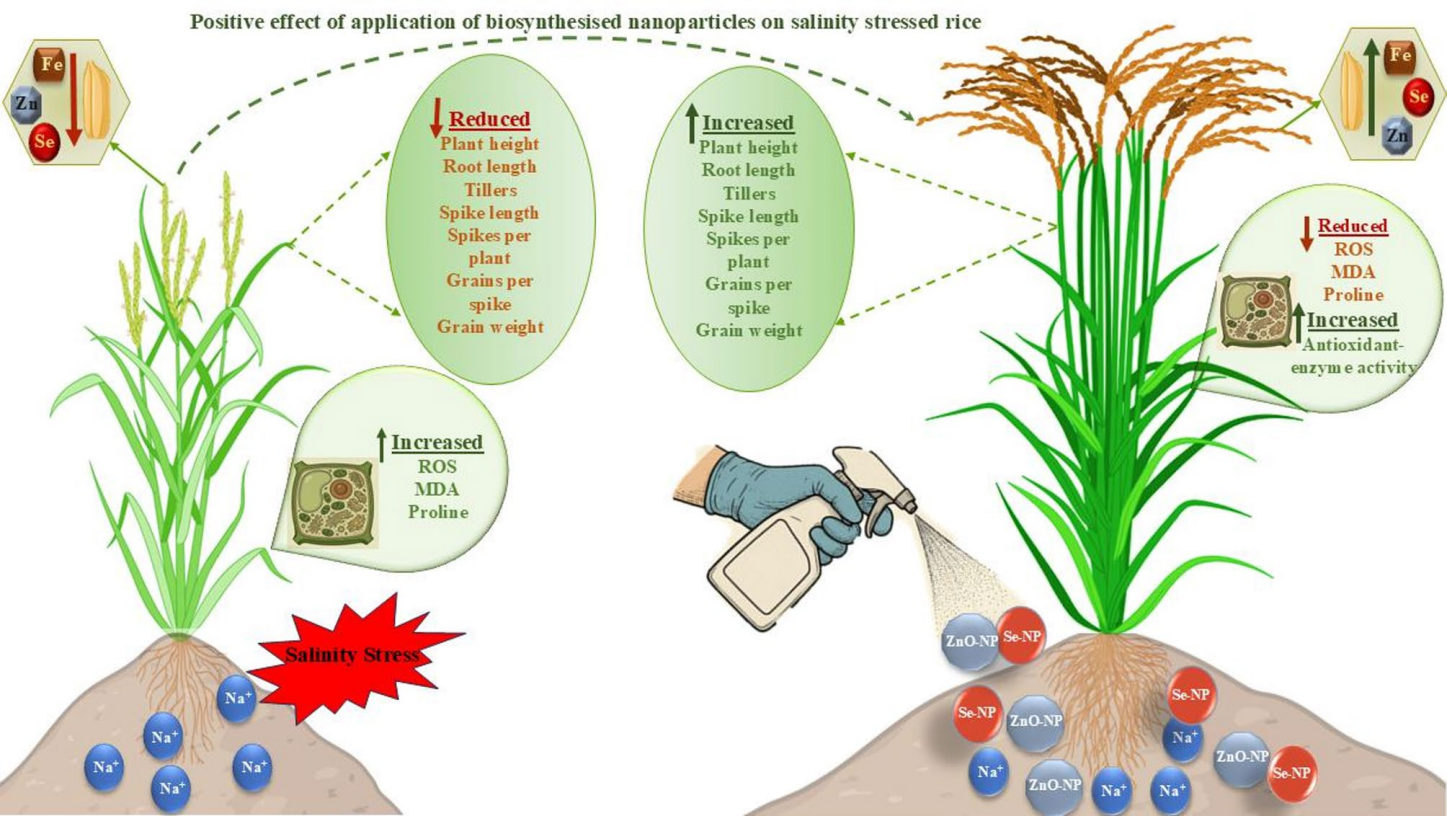
Effect of nanoparticle application on salinity-stressed rice.

Several studies have explored the role of Se-NPs and ZnO-NPs in crops, particularly in aspects such as growth and stress tolerance. Se-NPs (30 mg L-1) biosynthesised using grape extract significantly improved growth, antioxidant enzyme activity, and stress tolerance in mustard plants under high salinity conditions^14^. Similarly, the combined application of TiO_2_ NPs (100 mg L^− 1^) and chitosan-selenium NPs (20 mg L^− 1^) improved growth, photosynthesis, antioxidant activity, and glycoside (stevioside and rebaudioside A) content in *Stevia rebaudiana* under salinity stress, while reducing oxidative stress^15^. In safflower, the combined application of ZnO-NPs (17 mg L^− 1^) and Phytoguard (biofertilizer) markedly enhanced plant productivity, antioxidant activity, and key agronomic traits under saline conditions^16^. Positive effects were also observed in *Sorghum bicolor*, where seed priming with biosynthesized ZnO-NPs improved growth, ion balance, and antioxidant responses, while minimizing oxidative damage and restoring the anatomical integrity^17^. Additionally, foliar application of biosynthesised ZnO-NPs (50 mg L^− 1^) enhanced growth, pigment content, and nutrient uptake in *Vicia faba* under salinity stress^18^ (Fig. 1).

However, despite promising findings, most existing studies have focused on the individual effects of ZnO-NPs and Se-NPs under stressful conditions. There is a lack of comprehensive studies exploring their combined application in rice, particularly in saline environments. Understanding how these nanoparticles function together could reveal novel stress-mitigation mechanisms and open new avenues for nano-enabled sustainable rice farming. The novelty of this study lies in its focus on the combined application of Se-NPs and ZnO-NPs, an approach that is not well-documented in research. This combinatorial strategy is expected to offer synergistic benefits by targeting multiple pathways related to stress tolerance, ion homeostasis, and antioxidant defense. Therefore, the present study aims to investigate the individual and combined effects of green-synthesized Se-NPs and ZnO-NPs on the growth, physiological, biochemical, and yield-related traits of rice under salinity stress. This study is expected to make a significant and innovative contribution to the literature by addressing the following hypothesis: (a) the application of Se-NPs and ZnO-NPs will mitigate adverse effects of salinity stress on rice by improving growth, physiological responses, biochemical adaptations and yield parameters, (b) rice plants subjected to salinity stress and then subsequently treated with Se-NPs and ZnO-NPs will demonstrate elevated antioxidant enzyme activities and a concomitant reduction in oxidative stress markers relative to untreated plants, and (c) Se-NPs and ZnO-NPs will enhance ion homeostasis under salinity stress as indicated by increased proline accumulation, which helps maintain osmotic balance. The findings of this study will provide novel insights into nano-enabled strategies for enhancing rice performance in salt-affected soils, thereby contributing to sustainable and resilient agriculture.

## Materials & methods

### Plant material and seed Preparation

Rice seedlings of *Pusa Basmati 1* (PB1), as an elite, widely cultivated, salt-sensitive Indian Basmati rice cultivar, were collected for this experiment from the MNNIT Allahabad Department of Biotechnology. The rice seeds were washed more than 3 times with double-distilled water to remove dirt. They were then treated with 5% (v/v) sodium hypochlorite (NaOCl) solution and kept for 30 min to disinfect the seed coat from any bacteria or fungi growing on it. They were washed with sterilized water for four to five times to eliminate any residual sodium hypochlorite from the system. Subsequently, they were soaked in double-distilled water overnight to break their dormancy before sowing them in the pots.

### Nanoparticle biosynthesis and characterization

The Se-NPs and ZnO-NPs used in our study were green-synthesized in the laboratory following the protocols previously standardized and published by Mishra et al.^19^ and Afzal et al.^20^, respectively. Briefly, Se-NPs and ZnO-NPs were synthesized using aqueous leaf extract of *Hibiscus rosa-sinensis* and *Senna occidentalis*, respectively. For Se-NPs, sodium selenite (Na_2_SeO_3_) served as the precursor, was mixed with the plant extract, followed by incubation in optimized conditions. Similarly, ZnO nanoparticles (NPs) were prepared using zinc acetate under controlled conditions. Both nanoparticles were obtained in the size range of < 100 nm, as determined by scanning electron microscope (SEM), atomic force microscopy (AFM), and X-ray diffraction (XRD). SEM analysis revealed that Se-NPs were predominantly spherical, while ZnO-NPs had a roughly spherical and smooth surface morphology. AFM scans further confirmed these shapes. XRD patterns were recorded in the 2θ range of 20º-80º, which confirmed the crystalline nature of the nanoparticles. The crystallite size was calculated using the Debye-Scherrer equation. The synthesised nanoparticles were stable, well-dispersed, and consistent with previously reported green-synthesised NPs.

### Experimental design and plant husbandry

A pot experiment was conducted in the experimental field of the Department of Biotechnology, Motilal Nehru National Institute of Technology, Allahabad, India, during the rice growing season (June 24- October 24). The pilot study was conducted in a randomized block design with three replications for each. The experimental soil characteristics are listed in Table 1. The physical and chemical characteristics of soil were estimated using the protocol established by Walkley and Black^21^. The dimensions of pots were 25 cm in diameter and 30 cm deep, and each pot was filled with 10 kg of soil. A total of 25 seeds were sown initially in each pot. After 3 weeks, when seeds were germinated. The three-leaf plant stage was obtained, they were transplanted into another pot, and 3 hills per pot were maintained for better growth and performance.

**Table 1:** Initial characteristics of experimental soil.

Soil type	Texture	Ρb	pH	ECe (dSm^− 1^)	O.M. (%)	Zn (ppm)	Se (ppm)	Na^+^ (ppm)	K^+^ (ppm)
Non-saline soil	Sandy-loamy	1.54	7.51	1.85	0.84	123.3	0.31	285.3	130.7
Saline soil	Sandy-loamy	1.57	7.57	10.22	0.81	114.4	0.27	1423.2	131.9

Pb = bulk density; ECe = electrical conductivity of the soil saturation extract; O.M.= organic matter.

For salinity stress treatment, 300 mM NaCl, 150 mM CaCl_2,_ and 150 mM MgCl_2_ salts were mixed into the soil. Concurrently, NPs were applied to the plants using root-zone application. Applications began one week after transplanting and were carried out once a week for four consecutive weeks by directly adding the NPs to the root zone. This method ensured uniform exposure and facilitated efficient uptake of the nanoparticles under salinity-stressed conditions. In this study, the following NP treatments were applied: control plants (no treatment, C), stressed plants (salinity treatment, X), selenium nanoparticle-treated plants (S), zinc oxide nanoparticle-treated plants (Z), selenium + zinc oxide nanoparticle-treated plants (SZ), stressed + selenium nanoparticle-treated plants (SX), stressed + zinc oxide nanoparticle-treated plants (ZX) and stressed + selenium + zinc oxide nanoparticle-treated plants (SZX). Selenium nanoparticle was applied at a concentration of 10 mg kg-1 of soil (S & SX), zinc oxide nanoparticle at 25 mg kg-1 of soil (Z & ZX), and the combination of selenium and zinc oxide nanoparticle at 10 mg kg-1 and 20 mg kg-1 of soil (SZ & SZX), respectively. These concentrations were selected based on previously conducted studies that demonstrated effectiveness in rice germination^19,22^. NPK fertilizer was applied to the pots using aqueous solutions at a rate of 120:50:25 kg ha^-1^ (modified for pot size). Distilled water was regularly added to pots for irrigation to maintain the water-holding capacity (WHC) of the soil. The plants were grown in a controlled environment in a 12 h photoperiod with 70% relative humidity with the temperature of 28 °C. All biochemical and enzymatic assays were performed on leaf samples collected seven days after the final nanoparticle application, i.e., 30 days after the initiation of treatment.

### Measurement of plant morphological characters and yield

The study was conducted until the plants reached maturity (150 days after sowing). Three plants from each replication of the treatments, chosen at random, were used for the study. Seeds were harvested and the data on morphological attributes were recorded immediately afterwards. The following growth parameters were assessed for determining the rice yield after aforementioned treatments: plant height in cm (PH), root length in cm (RL), days to flowering (DF), number of tillers (TL), number of spikes per plant (SP), grains per spike (GS), spike length in cm (SL), fresh weight in g (FW), and 1000-grain weight in g (GW). For determining the plant height, plants were measured from the base at soil level to the tip of the tallest leaf using a flexible ruler. After completing the treatments, photographs of the rice plants were taken to document their growth and physiological response under stress conditions visually.

### Estimation of chlorophyll content, total protein and total sugar content

For estimation of photosynthesis activity, the chlorophyll content was determined using the established protocol by Lichtenthaler^23^. In a nutshell, 0.5 g of leaf sample was weighed and washed thoroughly, and then kept in 5 mL of 80% acetone in the dark overnight. The resulting solution was then centrifuged at 6000 rpm for 5 min, and the absorbance was recorded at 665 nm, 649 nm, and 480 nm for chlorophyll a, chlorophyll b, and carotenoids, respectively, in a BioTek Synergy H1 microplate Reader. The pigment content was expressed in mg.g^-1^FW.

To determine the protein concentration in the leaf tissue of mature rice plants, the method developed by Lowry et al.^24^ was employed. Initially, 100 mg of fresh leaf material was accurately weighed and homogenized in 5 mL of phosphate buffer (pH 7.3). The homogenate was then centrifuged at 5000 × g for 10 min at 4 °C to remove cellular debris. The supernatant was carefully transferred in a fresh microcentrifuge tube and stored at 4 °C for further analysis. For the assay, 100 µL of the supernatant was diluted to 1 mL with distilled water and 5 mL of Lowry reagent was added. After 30 min of incubation at room temperature, 0.5 mL of Folin-Ciocalteu (FC) reagent was added, followed by another 30 min of incubation in the dark. The absorbance was then measured at 650 nm. Protein content was determined using a standard curve generated from bovine serum albumin (BSA) and expressed as mg∙g^− 1^ FW.

The total soluble sugar content was estimated following the protocol of Hedge and Hofreiter^25^. Briefly, 100 mg of leaf sample was weighed and homogenized in 5 mL of 95% ethanol, followed by centrifugation at 5000×g for 10 min at 4 °C to remove cell debris. The supernatant was collected and kept in a refrigerator at 4 °C until future use. A 100 µL aliquot of the collected supernatant was diluted to 1 mL, and then 4 mL of Anthrone reagent was added. The mixture was then boiled in a water bath at 100 °C for 10 min, and the absorbance was recorded at 625 nm. The sugar content was estimated by plotting a standard curve using glucose and was expressed in mg/g FW.

### Determination of oxidant activity

Oxidant activity in plants under stress conditions was assessed through malonaldehyde assay (MDA) content analysis. To observe damage to the cell membrane, lipid peroxidation was estimated using MDA, following the protocol by Heath and Packer^26^ with slight modifications for enhanced accuracy. Approximately 100 mg of fresh leaf samples were weighed and homogenized in 1.5 mL of 0.1% (w/v) trichloroacetic acid (TCA) in a pre-chilled mortar and pestle to minimize oxidative changes during processing. In order to prevent enzymatic oxidation and to maintain the natural oxidative state, the chill state is necessary^27^.

The homogenate obtained was transferred to microcentrifuge tubes and centrifuged at 12,000×g for 15 min at 4 °C. This step helps to separate soluble oxidized compounds from debris. From the supernatant, 500 µL was carefully collected in a fresh 5 mL microcentrifuge tube. Then 1.5 mL of 0.5% (w/v) thiobarbituric acid (TBA) was added to it. The solution was mixed well using a vortexer^28^. The microcentrifuge tubes were then incubated in a hot water bath at 90 °C for 20 min to allow the formation of MDA-TBA complex. The reaction mixture was cooled immediately on ice to terminate the reaction. The cooled reaction mixture was centrifuged at 10,000×g for 5 min at 4 °C to remove any precipitated proteins that might interfere with absorbance. absorbance was recorded of the clear supernatant at 450, 532, and 600 nm using a spectrophotometer. MDA content was expressed in µmol mg^-1^ FW.

### Estimation of enzymatic and non-enzymatic antioxidant activity

After 30 days of treatment, the fresh rice leaves were collected, and the antioxidant activities of superoxide dismutase (SOD), ascorbic acid peroxidase (APX), catalase (CAT), and peroxidase (POD) were measured. The enzymes were extracted from the leaves using 50 mM ice-cold phosphate buffer saline (PBS) of pH 7.4, that contained 1 mM phenylmethylsulfonyl fluoride (PMSF) to inhibit proteases, 10 mM EDTA that chelated divalent cations to prevent enzyme degradation 8 mM MgCl_2_, and 1% (w/v) polyvinylpyrrolidone (PVP) to eliminate phenolics. The supernatant obtained after centrifugation at 12,000×g for 15 min at 4 °C was used to determine the activities of SOD, POD, APX, and CAT.

The Catalase (EC 1.11.1.6) activity was measured, using the method developed by of Aebi^13^ and improved by Hanif et al.^29,30^. Briefly, a reaction mixture of final volume 3 mL was prepared, containing 100 mM phosphate buffer (pH 7.4) and 15 mM of freshly prepared H_2_O_2_. The absorbance was measured at 240 nm for two minutes at 30-second intervals using a UV-visible spectrophotometer as soon as 100 µL of the enzyme extract was added to the reaction mixture. The molar extinction coefficient of H_2_O_2_ (39.4 M^− 1^ cm^− 1^) was used to evaluate further the enzyme activity, which was then expressed as µmol H_2_O_2_ oxidized min^− 1^ mg^− 1^ protein. APX (EC 1.11.1.11) activity was measured according to the protocol established by Nakano and Asada, with modifications as described by Sarkar and Oba^31,32^. APX was assessed in 1 mL of test mixture containing 2% (v/v) enzyme extracts, 0.5 mM of L-ascorbic acid, and 100 mM of phosphate buffer (pH 7.4). The amount of oxidized L-ascorbic acid at an absorbance of 290 nm. The activity was further calculated using the extinction coefficient for ascorbate: 2.8 mM⁻¹ cm⁻¹.

SOD (EC 1.15.1.1) activity was used to prevent the photochemical reduction of NBT, an approach developed by Beauchamp and Fridovich and refined by Giannopolitis and Ries, as well as more recently by Sanyal et al.^33–35^. SOD was measured in a reaction mixture containing 100 µL enzyme extract, 0.3 mM riboflavin, 1.3 mM bovine serum albumin, 15 mM NBT, and 10 mM EDTA^36^. Enzyme activity was assessed using absorbance measurements at 560 nm, which showed a 50% decrease in NBT. POD (EC 1.11.1.7) activity was measured using a reaction mixture that included 50 mM H_2_O_2_, 100 mM guaiacol, 100 µL of enzyme extract, and 100 mM phosphate buffer (pH 7.4). The absorbance was recorded at 420 nm over 2 min^37^. Proline was estimated as a potential non-enzymatic antioxidant, as previously established by Sarker & Oba^32^. A 3% (v/v) aqueous solution of sulfosalicylic acid was used to grind approximately 1 g of rice leaves. The homogenate was centrifuged at 12,000 × g for 15 min at 4 °C. Two mL of the obtained supernatant was mixed with equal amounts of glacial acetic acid and acidic ninhydrin at room temperature. This mixture was heated for 40 min at 95 °C, cooled on ice, and then 4 mL of toluene was added to extract the colored reaction product that had been separated from the aqueous phase. The toluene phase absorbance was read at 520 nm, and the buildup of free proline was assayed using the proline standard curve.

### Measurement of mineral content in grain

For determining the amount of zinc, selenium, iron, phosphorus. Potassium content in grain samples, 0.5 g of powdered rice sample was taken into a Teflon digestion vessel. A mixture of 5 mL of concentrated HNO₃ (trace-metal grade) and 1 mL of H₂O₂ was added. The sample was digested using a microwave digestion system at 200 °C for 30 min and then diluted to 50 mL with deionized water. The solution was filtered using a 0.45 μm membrane filter to remove particulates. Mineral content was then estimated using ICP-MS (Inductively Coupled Plasma-Mass Spectrometry) and certified reference materials (CRMs). NIST 1568b (rice flour) was used for method validation.

### Histological staining

For localization of O_2_.^-^ and H_2_O_2_ in rice leaf tissue, staining with Nitroblue tetrazolium chloride (NBT) and 3,3-diaminobenzidine (DAB), respectively, was performed. For NBT staining, the established protocol by Fryer et al.^38^ was utilized with a slight alteration. Briefly, rice leaves were submerged in a 0.1% solution of NBT and incubated at room temperature (25 °C) for 12 h. Subsequently, the leaves were immersed in boiling 90% ethanol for approximately 15 min to decolorize the leaves. The dark blue spots were observed as a result of the reaction between NBT and O_2_.^-^. For DAB staining, leaves were immersed in 0.1% DAB (pH 3.8) and incubated at room temperature (25 °C) for 12 h until brown precipitates were detected^39^. The leaves were bleached with boiling 96% ethanol (v/v). Subsequently, the leaves were photographed for record.

### Statistical analysis

To assess the significance of the experimental outcomes, a one-way analysis of variance (ANOVA) was performed following a completely randomized design. Differences among treatments and the control were analyzed using Tukey’s multiple range test to identify statistically significant variations. Data were presented as mean ± standard deviation, with each measurement conducted in a minimum of three independent replicates. A statistical significance threshold of *p* ≤ 0.05 was used, corresponding to a 95% confidence interval. For cluster heat map generation, we used SRplot web server (https://bioinformatics.com.cn/en)^40^.We also inspected pairwise Pearson’s correlations between the dependent variables in Python.

## Result

### Growth and yield response to salinity stress and the nanoparticle treatment

**Fig. 2:**
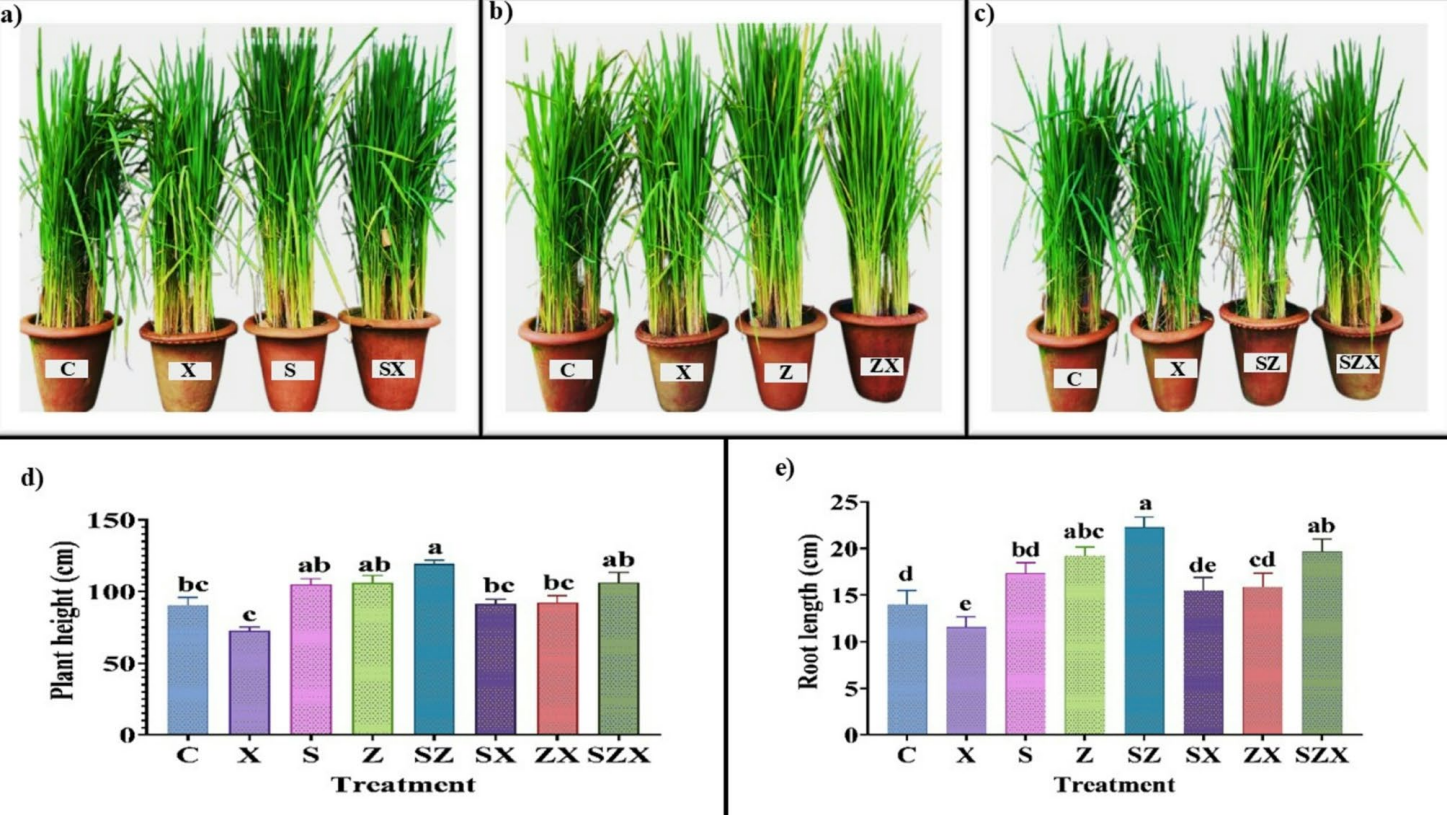
Effect of nanoparticle application on morphological characteristics of rice plant under different treatment conditions **(a)** Illustration of the effect of varied salinity stress and its mitigation using Se-NPs (**b)** Pictorial representation of rice plant response to salinity stress with ZnO-NPs application **(c)** Figure depicting the morphological differences in rice plant under salinity stress and its amelioration after combined application of Se-NPs and ZnO-NPs. **(d)** Graph showing the effect of varied salinity stress and subsequent nanoparticle treatment on plant height (cm). (**e**) Graph of root length (in cm) of stressed and nanoparticle-treated rice plants. The data presented is means of three replicates containing three hills per pot each ± standard error of means. Bars followed by alphabets represent the significance levels according to Tukey’s multiple range test (abbreviations for each treatment are defined in Materials and Methods section).

Plant growth and the associated morphological parameters are strongly influenced by the plant’s tolerance to abiotic stresses such as salinity and chemical characteristics of salt-affected soils. In our study, analysis of variance (ANOVA) indicated statistically significant differences (*p* ≤ 0.05) across all measured parameters when comparing salinity-stressed rice plants with those treated with Se-NPs, ZnO-NPs, and their combined application (Fig. 2a-c).

Compared to the control plants, rice growth exhibited a notable decline when exposed to salinity stress alone. Specifically, PH and RL were significantly affected and decreased by 19.8% and 17.64% respectively. The average PH under salinity stress was recorded at 72.67 ± 2.51 cm, while control plants reached 90 ± 5.13 cm (Fig. 2d). Similarly, RL declined from 14 ± 1.5 to 11.53 ± 1.13 cm in stressed plants **(**Fig. 2e**)**.

**Fig. 3:**
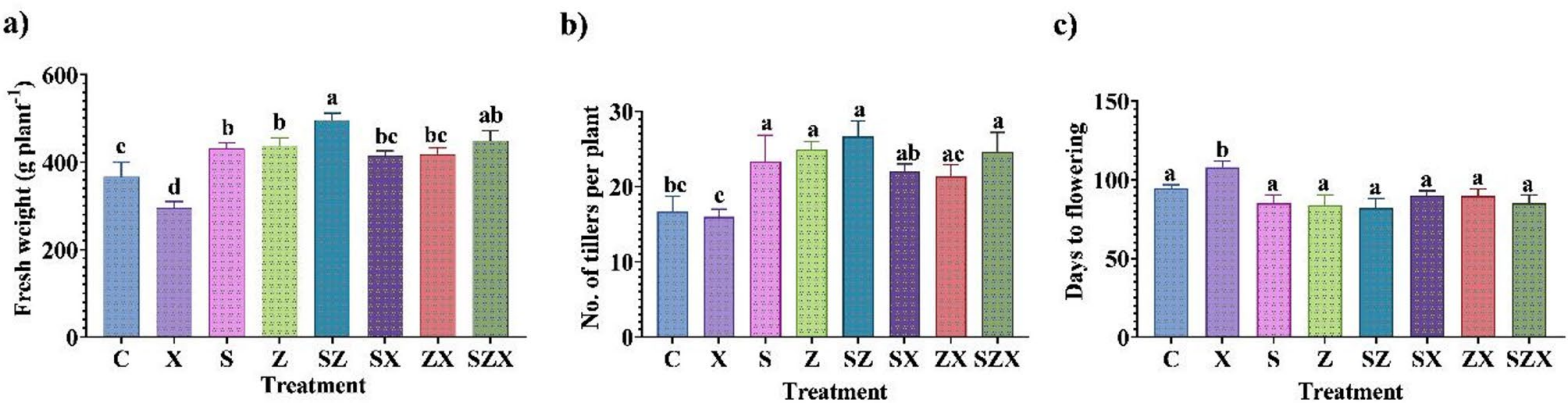
Agronomic parameters of rice plants harvested after nanoparticle treatment under salinity-stressed and non-stressed conditions **(a)** Graph representing fresh weight (g plant^− 1^) of untreated and treated rice plants (**b)** Graph of the number of tillers in the rice plants under salinity stress and after nanoparticle application **(c)** Graph showing the number of days to flowering for the salinity-stressed plants and the nanoparticle-treated plants. The data presented is the mean of three replicates containing three hills per pot, each ± standard error of means. Bars followed by alphabets represent the significance levels according to Tukey’s multiple range test (abbreviations for each treatment are defined in the Materials and Methods section).

FW and TL also showed reduction of 4% and 19.59% respectively, under salt stress (Fig. 3a–b). Furthermore, flowering was delayed in stressed plants, with an average onset of 71.8 ± 3.6 days, indicating a clear disruption in developmental timing due to saline soil conditions (Fig. 3c).

However, the application of NPs markedly improved growth and morphological traits, even under salinity stress. Treatment with Se-NPs (SX) significantly enhanced PH, RL, TL and FW by 25.68%, 34.40%, 33.50% and 40.48% respectively, compared to untreated stressed plants (X). Similarly, ZnO-NPs treatment (ZX) increased these parameters: PH, RL, TL, and FW by 23.39%, 37.58%, 33.34% and 41.46%, respectively. These results indicate that both nanoparticles individually improved plant performance under stress, restoring growth to levels comparable to those of non-stressed control plants.

Notably, the combinatorial application of Se-NPs and ZnO-NPs yielded the most pronounced improvement in the growth traits. Compared to salinity stressed plants (X), PH, RL, TL, and FW increased by 46.32%,70.53%, 54.16% and 52.35% respectively. Moreover, in some parameters (such as RL and FW) values in the combined treatment group even exceeded those of the control, suggesting a potential synergistic effect.

**Fig. 4:**
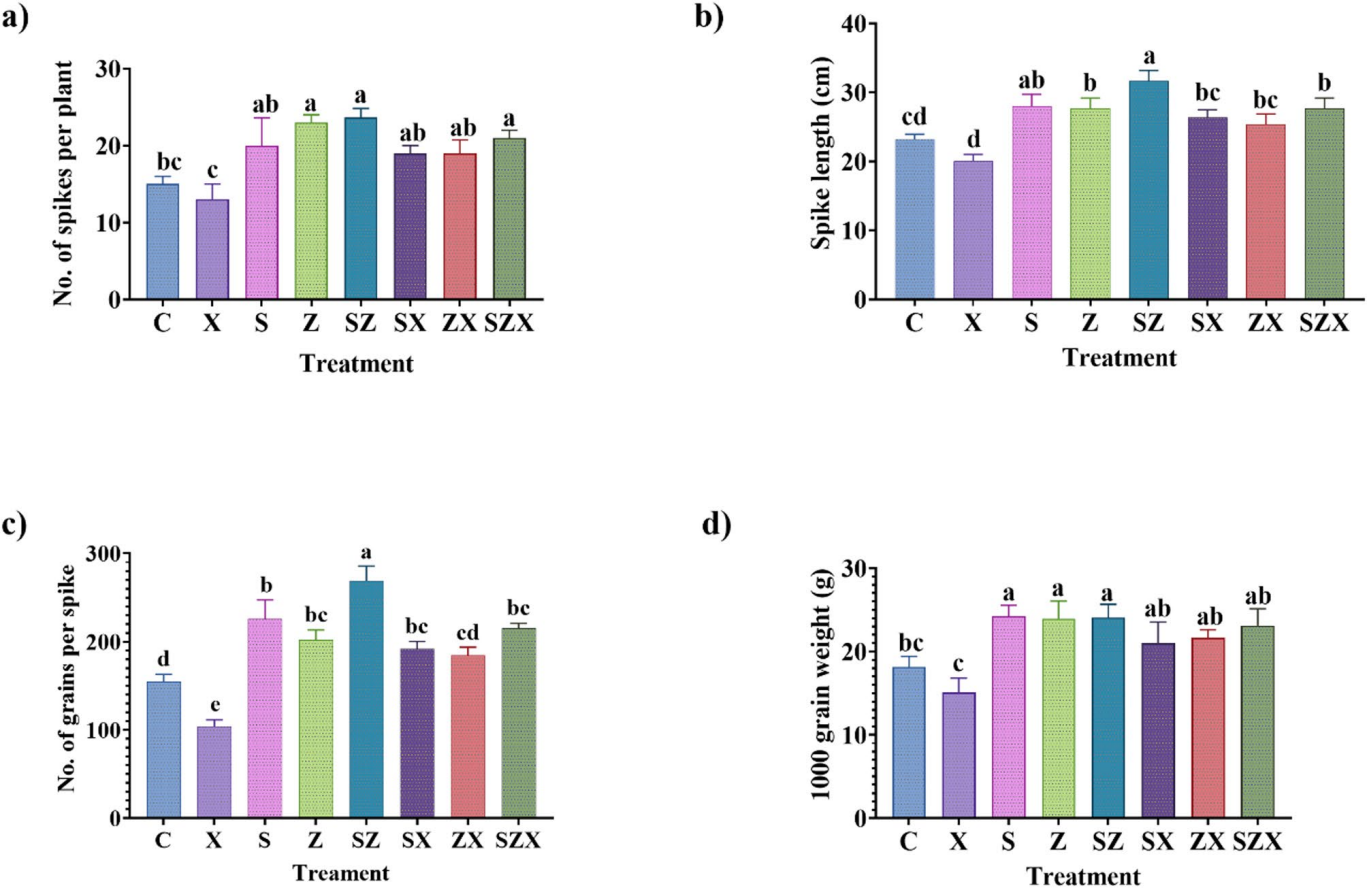
Yield characters of rice plants harvested after complete maturation (**a)** Graph showing no. of spikes per plant (**b)** Graph representing spike length (in cm) (**c)** Graph representing no. of grains per spike (**d)** Graph representing 1000-grain weight (in grams). The data presented is the mean of three replicates containing three hills per pot, each ± standard error of means. Bars followed by alphabets represent the significance levels according to Tukey’s multiple range test (abbreviations for each treatment are defined in Materials and Methods section).

Salinity stress also had a pronounced adverse effect on yield-related traits, with reductions observed in SP by 13.34%, SL by 13.66%, GS by 32.96%, and GW by 16.69% as compared to the control (Fig. 4a-d).

Soil application of Se-NPs and ZnO-NPs under saline conditions effectively mitigated these adverse effects. Treatment with Se-NPs increased by 46.15% (SP), 31.66% (SL), 84.86% (GS), and 39.2% (GW) over the salinity-stressed plants. Similarly, ZnO-NPs enhanced the same parameters: SP, SL, GS, and GW by 46.15%, 26.66%, 78.1% and 43.19%, respectively (Fig. 4a-d). These improvements brought yield-related traits closer to, or in some cases above, control levels, particularly for GS and GW.

Notably, the combined application of Se-NPs and ZnO-NPs produced the most significant improvements, with an increase of 61.53% in SP, 38.33% in SL, 100.7% in GS, and 52.89% in GW under salinity conditions. Significantly, these enhancements not only mitigated the effects of salinity but, in some traits, outperformed the unstressed control group (C), underscoring the potential synergistic role of combined NP s in improving both vegetative and reproductive parameters under stress.

Our results show the synergistic effect of the combined nanoparticle treatment, which outperformed individual applications in mitigating the negative impact of salinity on rice growth. These results also support the existing literature, which suggests that Se-NPs and ZnO-NPs promote plant growth and productivity under salinity stress conditions. Under non-saline conditions, nanoparticle treatments also promoted growth as significant differences in morphological and yield traits were observed between the control and treated groups, demonstrating that Se-NPs and ZnO-NPs have intrinsic growth-promoting properties. The overall trend of effectiveness under salinity stress followed the order: SZX > SX = ZX.

In conclusion, salinity stress substantially impairs rice growth and morphological development. However, the application of NPs, particularly the combined use of Se-NPs and ZnO-NPs, significantly alleviates these adverse effects. The interaction effects between salinity stress and nanoparticle treatments were statistically significant for all measured parameters, highlighting the potential of nanoparticle-based strategies to enhance crop performance under abiotic stress conditions. Therefore, from an agronomic perspective, both Se-NPs and ZnO-NPs can be considered effective and safe nano-fertilizers for improving crop performance under adverse environments.

### Pigment, total protein and total sugar content in salinity Stressed-Nanoparticle treated plants

**Fig. 5:**
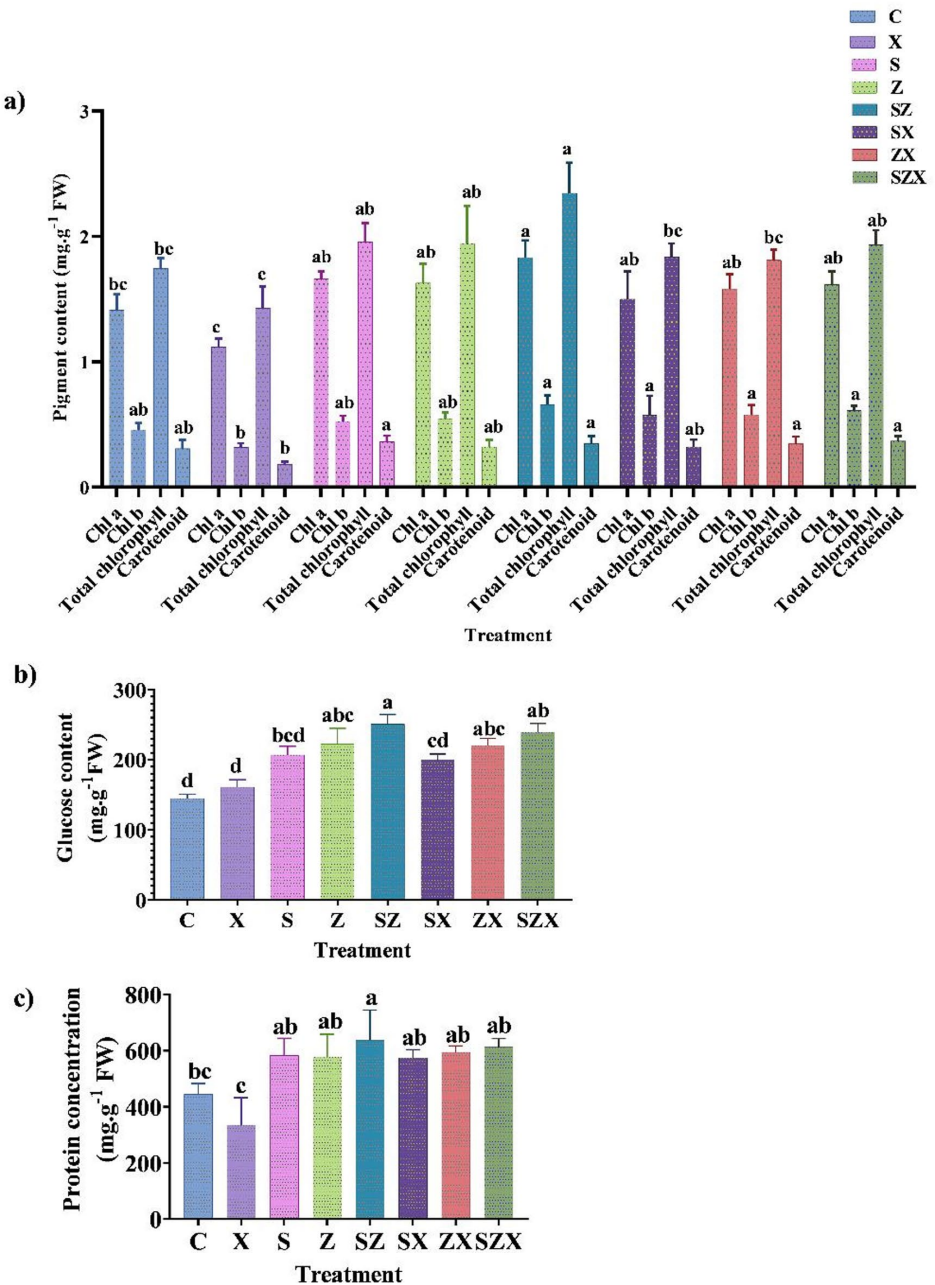
Biochemical characteristics of rice plant under different treatment conditions **(a)** Graph showing effect of varied salinity stress and subsequent nanoparticle treatment on pigment content (mg.g^− 1^FW) (**b)** Graph of glucose content (in mg.g^− 1^FW) of stressed and nanoparticle treated rice plant (**c)** Graph of protein content (mg.g^− 1^FW) in the rice plants under salinity stress and after nanoparticle application. The data presented is the mean of three replicates containing three hills per pot, each ± standard error of means. Bars followed by alphabets represent the significance levels according to Tukey’s multiple range test (abbreviations for each treatment are defined in Materials and Methods section).

Salt tolerance in plants is a complex trait that is influenced by physiological and biochemical processes. Under salinity stress, photosynthetic efficiency was significantly impaired, likely due to reduced gaseous exchange and inhibition of carbon fixation enzymes within the chloroplast. This was accompanied by a marked decline in chlorophyll and other pigment content in the rice plants. The chlorophyll a (Chl a), chlorophyll b (Chl b), total chlorophyll (total Ch), and carotenoid content were significantly reduced in salinity-stressed plants by 20.8%, 23.46%, 18.15%, and 40.77% respectively, when compared to the control. However, subsequent nanoparticle treatments resulted in a notable recovery of these pigments. Specifically, application of Se-NPs + ZnO-NPs to salinity stressed rice plants resulted in significant increase in Chl a, Chl b, total Ch and carotenoid content by 44.44%, 74.85%, 35.38%, and 102.1% respectively, when compared to the salinity stressed plants without treatment (Fig. 5a). Treatment with Se-NPs alone enhanced Chl a, Chl b, total Ch and carotenoids content by 34.2%, 64%, 29.71% and 89.38%, respectively **(**Fig. 5a**)**. Similarly, ZnO-NPs treatment helped recover Chl a, Chl b, total Ch and carotenoids content by 41.4%, 65.5%, 26.71%, respectively, compared to untreated stressed sets.

Furthermore, stressed rice plants exhibited a notable physiological response in terms of total sugar accumulation and protein degradation. The total sugar content was slightly increased in stressed plants (X) by 11.34% compared to the unstressed control (C), likely reflecting an adaptive response to meet the higher energy demands under stress conditions. The combined application of Se-NPs and ZnO-NPs, however, led to a significant increase in the total soluble sugar content by 48.57% as compared to stressed rice plants (Fig. 5b). Individually, Se-NPs and ZnO-NPs also promoted sugar accumulation, with increase of 23.69% and 36.82% respectively, in stressed plants, relative to untreated stressed plants.

In contrast, salinity stress adversely affected protein metabolism. In the stressed plants a 24.12% reduction in total protein content was observed compared to their unstressed counterpart (C) indicating possible denaturation of proteins and inhibition of enzyme activity under stress (Fig. 5c). However, treatment with NPs effectively mitigated this decline. The individual treatment with Se-NPs and ZnO-NPs helped restore total protein levels by 71.6% and 77.75% respectively, compared to the stressed rice plants. The combined application of Se-NPs + ZnO-NPs was even more effective in mitigating the stress and has significantly enhanced the protein content by 83.40% compared to the stressed rice plants (Fig. 5c). These findings suggest that the combined application of SeNPs and ZnO-NPs may serve as a promising strategy to alleviate the detrimental effects of salinity stress by enhancing the biochemical profile of plants.

### Effect on lipid peroxidation due to salinity stress and its mitigation using NPs

**Fig. 6:**
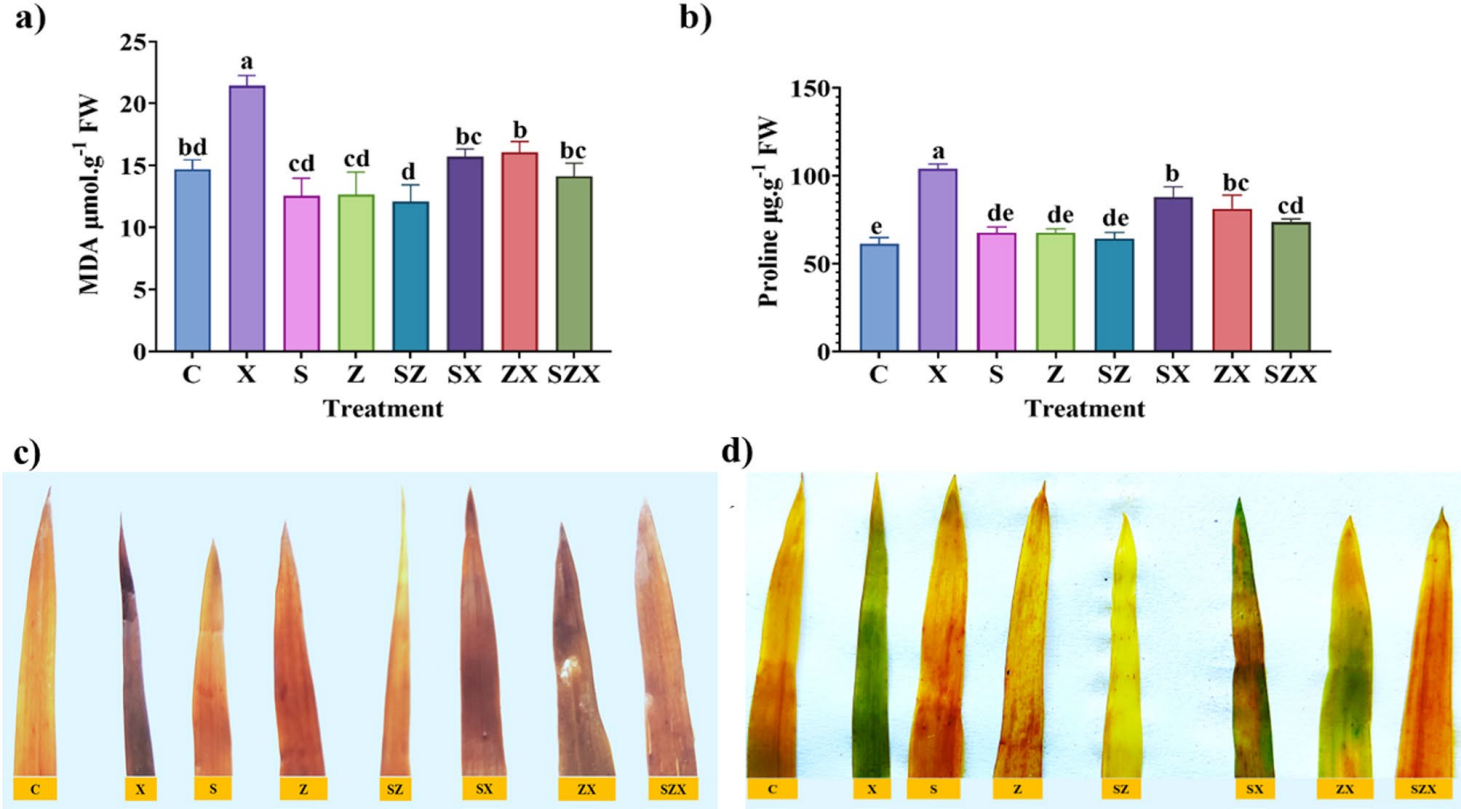
The effect of nanoparticle treatment on stressed plants and their non-enzymatic antioxidant defense mechanisms in plants. **(a)** Graph representing malondialdehyde (MDA) levels as a measure of lipid peroxidation (**b)** Graph depicting proline content in plants after nanoparticle treatment. (**c)** Detection of hydrogen peroxide (H_₂_O_₂_) via 3,3’-diaminobenzidine (DAB) staining. The brown coloration indicates the presence of H_₂_O_₂_, reflecting the extent of oxidative stress and the effect of subsequent nanoparticle treatment on reactive oxygen species (ROS) accumulation in rice leaves. (**d)** Visualization of superoxide production using nitro blue tetrazolium (NBT) staining to assess free radical generation in rice leaves after nanoparticle application both individual treatments and in combination. The intensity of blue color represents the intensity of superoxide generation under various treatment conditions. The data represent the mean values of three independent replicates, each containing three hills per pot, with error bars indicating the standard error of the mean. Bars annotated with different letters indicate statistically significant differences, as determined by Tukey’s multiple range test (treatment abbreviations are provided in the Materials and Methods section).

Membrane lipid peroxidation, which is assessed through MDA content, was observed to increase by 45.98% in stressed rice plants compared to that of the control plants, indicating enhanced oxidative damage under stress conditions. Treatment with Se-NPs and ZnO-NPs individually demonstrated a mitigating effect, reducing the MDA levels by 26.66% and 24.94%, respectively, relative to stressed plants (Fig. 6a). Although the MDA levels in Se-NPs and ZnO-NPs treated stressed plants remained slightly elevated compared to the control by 7% and 6.55% respectively. When compared with MDA content, Se-NPs and ZnO-NPs showed a reduction. The combined application of Se-NPs and ZnO-NPs resulted in a more pronounced reduction in lipid peroxidation, with MDA content 3.79% higher than the control and 34.09% lower than in stressed plants. It was found that the Se-NPs and ZnO-NPs treatments can offer protective effects in mitigating stress. These results suggest the potential for these NPs in open-field applications aimed at mitigating salinity-induced oxidative damage in crops.

### Proline content in the stressed plants

Proline plays a crucial role in plant stress responses, functioning both as an osmoprotectant and a physiological indicator of stress severity. Our results showed that salinity stress induced a significant increase in proline content by 69.69% compared to the control, highlighting its role in stabilizing proteins and membranes, maintaining osmotic balance, and scavenging reactive oxygen species (ROS). However, in rice plants treated with Se-NPs and ZnO-NPs under salinity stress, the proline content was 43.36% and 32.43%, respectively, when compared to the control. The combined treatment with Se-NPs + ZnO-NPs resulted in a more moderate increase of 20.23% compared to the control. The 20.23% increased proline content was found. When evaluated against stressed plants, these treatments led to a reduction in proline content by 15.5%, 21.95% and 29.13% for Se-NPs, ZnO-NPs and Se-NPs + ZnO-NPs, respectively (Fig. 6b). These findings indicate that the nanoparticle treatments were effective in mitigating salinity-induced stress, thereby reducing the plant’s reliance on proline accumulation. Thus, proline not only acts as a biochemical defense molecule but also as a reliable biomarker for assessing stress intensity and the efficacy of protective treatments.

### Histochemical staining for detection of ROS

Histochemical staining by DAB and NBT demonstrated a notable reduction in the accumulation of superoxide anion (O_2_^-^) and hydrogen peroxide H_2_O_2_ in rice plants treated with Se-NPs and ZnO-NPs under salinity stress, compared to untreated stressed plants (Fig. 6c-d). This observation suggests that the nanoparticle treatments effectively reduced reactive ROS levels under stress conditions. As ROS level during stress conditions is tightly regulated by the homeostasis between their generation and scavenging, the reduced ROS levels in nanoparticle-treated plants indicate an enhanced activation of the antioxidant defense mechanism. These results suggest that the combined and individual application of Se-NPs and ZnO-NPs improved ROS detoxification capacity, likely through the upregulation of antioxidant enzymes, thereby protecting against oxidative damage under salinity stress.

### Anti-oxidant enzyme activity

**Fig. 7:**
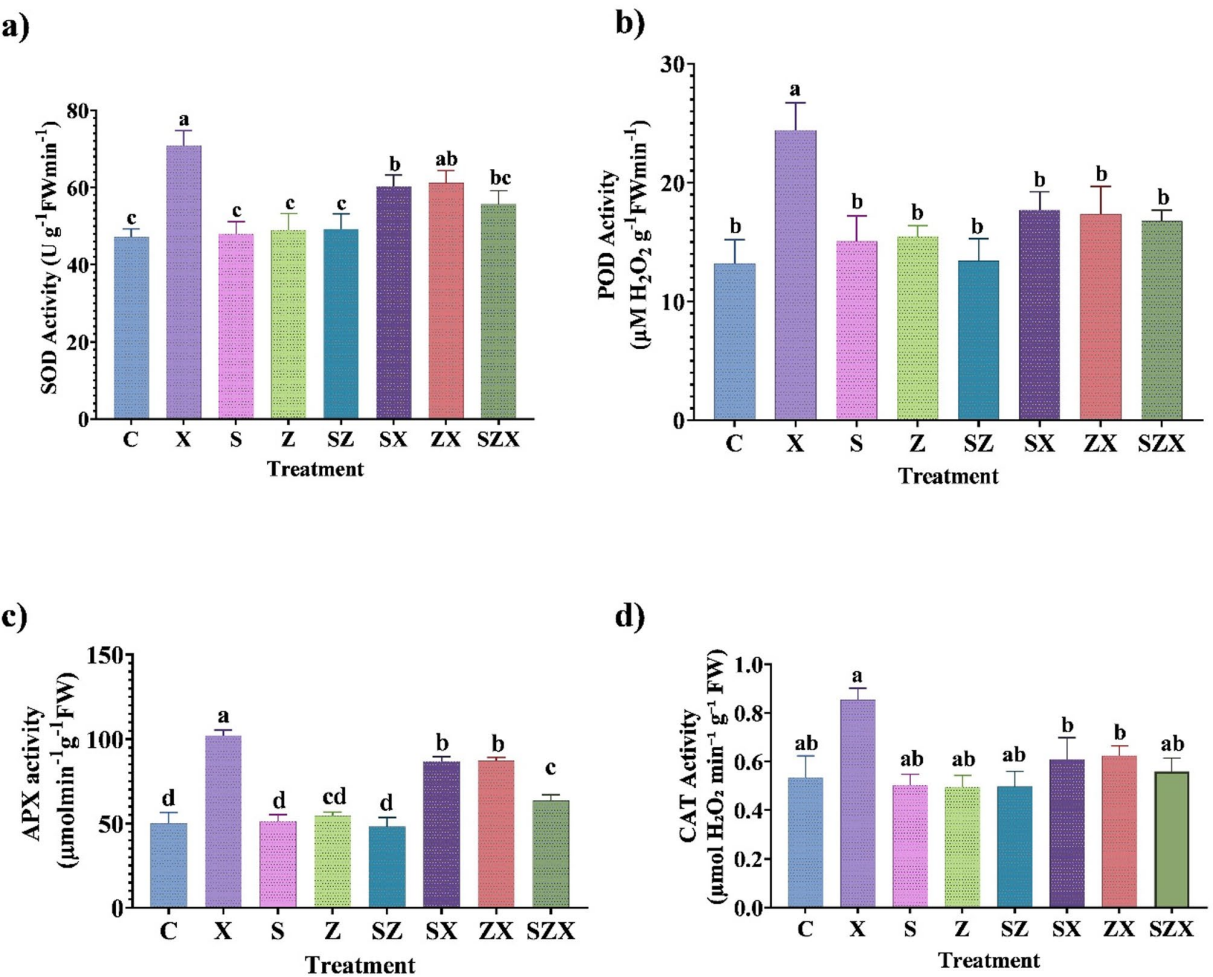
The effect of nanoparticles on enzymatic antioxidant defense mechanisms in stressed plants. **(a)** Graph showing the effect of various nanoparticle treatments on superoxide dismutase (SOD) activity in salinity stressed plants. (**b)** Graph illustrating the changes in peroxidase (POD) activity in stressed and non-stressed plants. (**c)** Graph depicting ascorbate peroxidase (APX) activity in treated plants. **(d)** Graph depicting catalase (CAT) activity levels in treated plants. The data represent the mean values of three independent replicates, each containing three hills per pot, with error bars indicating the standard error of the mean. Bars annotated with different letters indicate statistically significant differences, as determined by Tukey’s multiple range test (treatment abbreviations are provided in the Materials and Methods section).

The salinity stress induced the activity of antioxidant enzymes in rice plants, with increases of 50.06% in SOD, 85% in POD, 104.28% in APX, and 59.92% in CAT as compared to the control (Fig. 7a-d). The activities of SOD, POD, APX, and CAT were increased by all nanoparticle treatments under salt-stress conditions. Treatment with Se-NPs resulted in enhanced activity of SOD (27.63%), POD (34.2%), APX (73.42%) and CAT (14.33%) relative to the control. Similarly, with ZnO-NPs treatments, an increase in the activity of SOD, POD, APX, and CAT was observed by 29.58%, 31.61%, 74.28%, and 17.16%, respectively, relative to the control condition. The combined application of Se-NPs + ZnO-NPs also recorded a relatively increased SOD, POD, APX and CAT activity by 18.06%, 26.92%, 27.14% and 4.7% respectively, as compared to control. Although these values were slightly lower than those observed in untreated stressed plants, this trend indicates a reduced stress load resulting from the nanoparticle treatment. The decline in enzyme activity relative to stressed plants suggests that the treatments effectively mitigated oxidative stress, reducing the demand for elevated antioxidant responses. These results demonstrate that nanoparticle applications positively influence antioxidant defense mechanisms and can be considered effective in enhancing enzymatic antioxidant activity under both stress and non-stress conditions.

### Effect of nanoparticle treatment on mineral accumulation in rice grains

The impact of Se-NPs and ZnO-NPs biofortification on the accumulation of mineral elements in rice grains was assessed under both salinity-stressed and non-stressed conditions. As shown in Table 2, the control rice plants exhibited baseline concentrations of Se (0.17 ± 0.01 ppm), Zn (25 ± 0.55 ppm), Fe (6.6 ± 0.36 ppm), P (282.89 ± 3 ppm), and K (250.2 ± 1.7 ppm). Under salinity stress, application of Se-NPs alone led to a significant increase in Se accumulation in rice grains (S: 0.73 ± 0.02 ppm). However, co-application with salinity resulted in comparatively lower Se levels (SX: 0.44 ± 0.04 ppm). However, Se-NPs treatment under stress did not result in notable changes in the concentrations of Fe (SX: 6.4 ± 0.4 ppm), Zn (SX: 23.3 ± 0.8 ppm), K (SX: 221 ± 2.2 ppm), and P (SX: 216.3 ± 2.4 ppm), indicating a limited influence on overall nutrient uptake. In contrast, ZnO-NPs application significantly enhanced nutrient accumulation under salinity stress, with increased concentrations of Se (0.26 ± 0.05 ppm), P (221.3 ± 1.9 ppm), K (215.6 ± 2.1 ppm), Zn (28.9 ± 1.3 ppm), and Fe (7.3 ± 0.32 ppm). This suggests that ZnO-NPs may play a crucial role in mitigating salt-induced impairment of nutrient uptake and maintaining mineral homeostasis in rice plants. Moreover, combined treatment with Se-NPs and ZnO-NPs under salinity stress further supported mineral accumulation although differences were not statistically significant. The concentrations recorded were Se (0.57 ± 0.03 ppm), P (216.3 ± 2.9 ppm), K (241.5 ± 3.4 ppm), Zn (31.8 ± 0.9 ppm), and Fe (7.4 ± 0.29 ppm). These results indicate a potential synergistic effect of Se and Zn biofortification in enhancing quality under salinity stress.

**Table 2:** Grain mineral content of different treatment samples of rice plants. Data are average values for 3 replicates ± standard deviation (SD).

Minerals (ppm)	LOQ (ppm)	Treatments							
		C	X	S	Z	SZ	SX	ZX	SZX
Se	0.02	0.17 ± 0.01	0.08 ± 0.02	0.73 ± 0.02	0.25 ± 0.03	0.64 ± 0.05	0.44 ± 0.04	0.26 ± 0.05	0.57 ± 0.03
Zn	0.02	25 ± 0.55	18.5 ± 1.15	28.1 ± 1.5	34.8 ± 1.2	34.7 ± 1.6	23.3 ± 0.8	28.9 ± 1.3	31.8 ± 0.9
Fe	0.05	6.6 ± 0.36	4.4 ± 0.5	6.7 ± 0.35	7.6 ± 0.5	7.1 ± 0.6	6.4 ± 0.4	7.3 ± 0.32	7.4 ± 0.29
K	0.05	250.2 ± 1.7	188.1 ± 1.4	234.6 ± 4.5	272.3 ± 2.4	252.4 ± 2.6	221 ± 2.2	215.6 ± 2.1	241.5 ± 3.4
P	0.02	282.89 ± 3	210.8 ± 1.75	284.6 ± 3.5	287.9 ± 2.2	285.4 ± 2.7	216.3 ± 2.4	221.3 ± 1.9	216.3 ± 2.9

LOQ = Limit of Quantification.

### Relationship among various morpho-physicochemical parameters in rice grains

Under salinity stress, strong positive correlations emerged among growth parameters, with PH showing particularly robust relationships with SL, RL, and total Ch (*r* = 0.97, *p* < 0.001). TL correlated strongly with SP (*r* = 0.98, *p* < 0.001), reflecting their shared dependence on axillary bud activation. GW correlated significantly with both DF (*r* = 0.97, *p* < 0.001), FW and protein (*r* = 0.94, *p* < 0.001), and Chl a (*r* = 0.95), indicating that delayed flowering may compromise yield components.

**Fig. 8:**
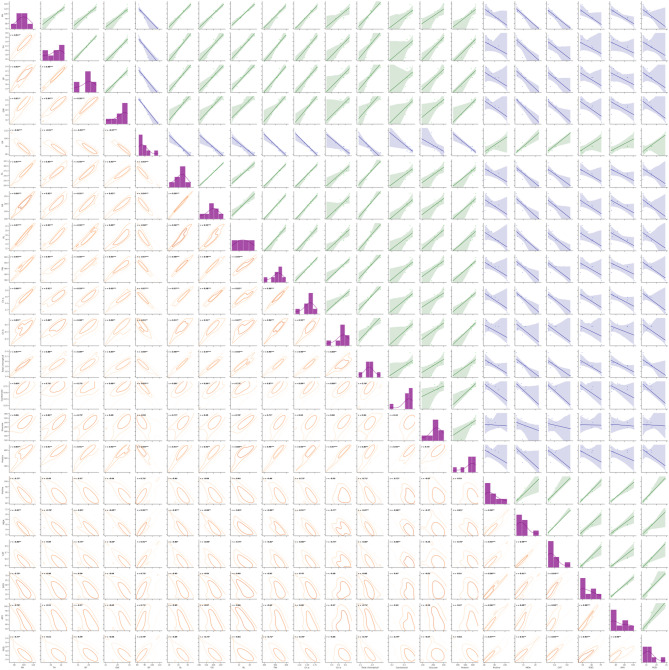
Pairwise correlation matrix of salinity stress response parameters. Upper triangle: Scatter plots with regression lines (green = positive relationships, blue = negative relationships). Diagonal: Histograms with kernel density estimates showing variable distributions. Lower triangle: Bivariate density plots with Pearson correlation coefficients (*p < 0.05, **p < 0.01, ***p < 0.001). Plant height (PH), days to flowering (DF), number of tillers (TL), number of spikes per plant (SP), grains per spike (GS), spike length (SL), fresh weight (FW), 1000-grain weight (GW), and root length (RL).

Oxidative stress markers exhibited expected negative correlations with antioxidant enzymes (Fig. 8). MDA content showed positive relationship with CAT activity (*r* = 0.99, *p* < 0.001), SOD activity correlated positively with proline accumulation (*r* = 0.98, *p* < 0.001), supporting proline’s role in ROS scavenging. The strong positive correlation between CAT and SOD activities (*r* = 0.94, *p* < 0.001), POD and CAT activities (*r* = 0.95, *p* < 0.001), SOD and APX activities (*r* = 0.98, *p* < 0.001) suggests coordinated enzymatic defense mechanisms. Notably, Chl a demonstrated a stronger relationship with growth parameters (*r* = 0.91–0.98) than Chl b (*r* = 0.85–0.96). Total Ch content correlated significantly with carotenoid levels (*r* = 0.76, *p* < 0.05), indicating parallel degradation of photosynthetic pigments under salinity stress.

## Discussion

In recent years, the stress. Application of nanotechnology in agriculture has expanded dramatically, particularly in strategy aimed at enhancing tolerance to abiotic stress, such as salinity. Zinc (Zn) is an essential micronutrient required by all organisms, including plants, where it plays a critical role in various physiological and biochemical processes^41^. It contributes to enzyme activation, cell division, nitrogen metabolism, photosynthetic pigment synthesis, and the maintenance of membrane integrity and phospholipid structure^42^. Although required in trace amounts, zinc deficiency can severely affect plant growth and productivity^43^. Selenium (Se), though considered a non-essential but beneficial element in plants, has shown positive effects at lower concentrations^44,45^. It acts as an antioxidant, supports plant growth, and protects against membrane lipid peroxidation^46,47^. However, at higher concentrations, Se may exhibit prooxidative propertiesleading to negative effects on yield^48^. Additionally, Se influences chlorophyll content and is associated with several health-promoting properties, including antioxidative, anticancer and antiaging effects^44^.

**Fig. 9:**
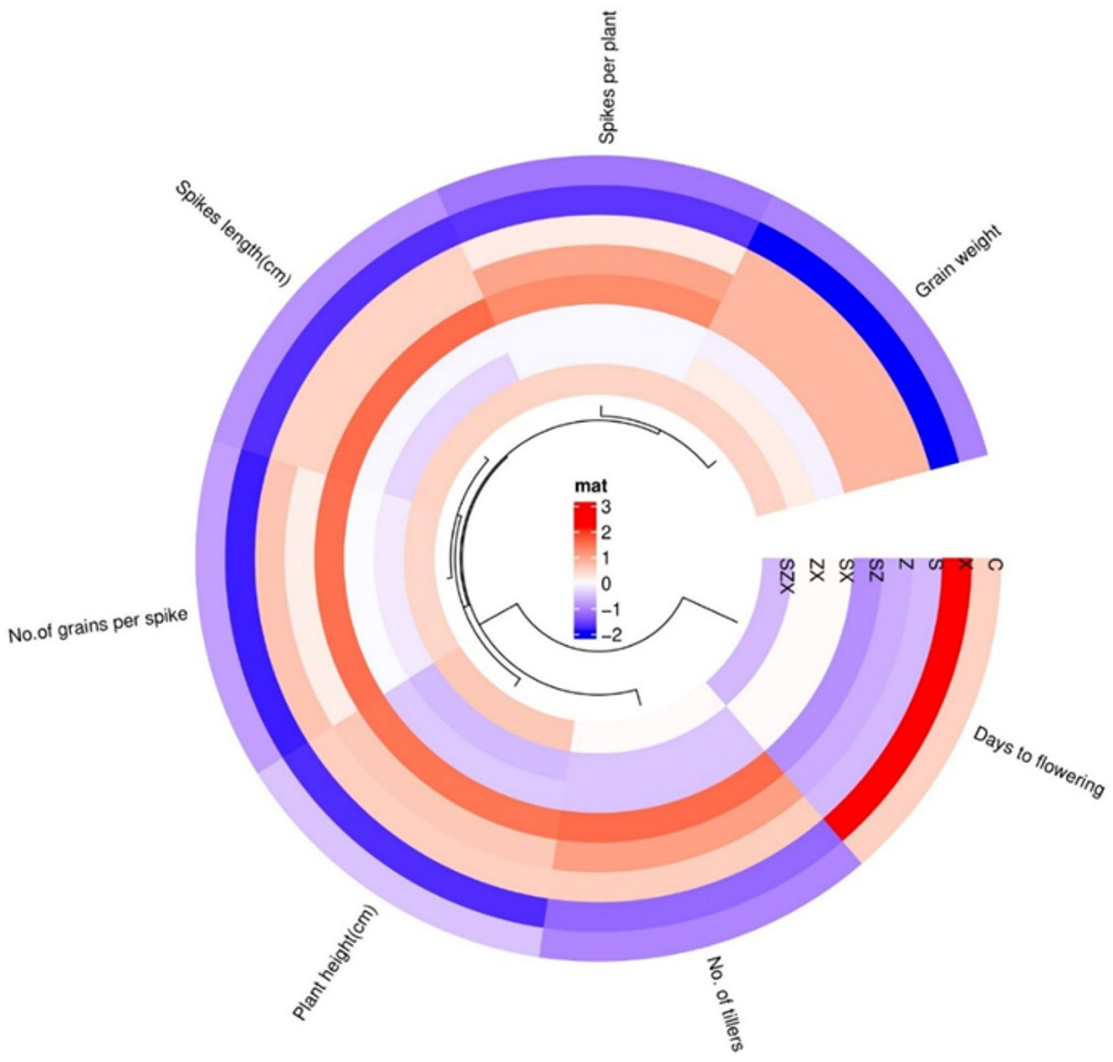
The cluster heat map depicting the correlation among various yield-related traits under the different treatment conditions. Each concentric circle represents a distinct experimental treatment (C, X, S, Z, SZ, SX, ZX, & SZX). At the same time, the radial arcs denote the individual agronomic traits assessed (plant height, no. of tillers, days to flowering, spike length, no. of spikes per plant, no. of grains per spike, & thousand-grain weight). The heatmap uses a color gradient scale which illustrates the relative intensity of each trait, with red indicating the highest value and blue representing the lowest. This visual representation facilitates the comparative evaluation of trait performance and interaction patterns in response to various treatments.

Salt stress inhibits the uptake of mineral nutrients, leading to nutrient deficiency and stunted growth in rice^49^. Root systems are particularly vulnerable, as salinity significantly impairs root elongation, likely due to cellular sensitivity to salt or altered water availability^50^. In the present study, green-synthesized Se-NPs and ZnO-NPs applied as nano-fertilizers significantly enhanced morphological, biochemical, and physiological parameters under salinity-stressed and non-stressed conditions, corroborating earlier studies^51–53^. Key growth metrics, including plant height, root length, biomass, tiller number, and yield, showed notable improvements under nanoparticle treatment. Tiller number, a crucial yield determinant, was positively affected, with higher tiller counts correlating with increased grain yield^54^. Cluster heatmap analysis (Fig. 9) further highlighted a strong positive correlation among PH, GS, SL, GW, and TL. Conversely, these traits exhibited a significant negative correlation with days to flowering, suggesting that delayed flowering can suppress yield. Previous studies support these findings, indicating that traits like PH and TL are often interlinked with yield components across various crops, including wheat and barley^55,56^. In dwarf waxy rice, plant height is positively correlated with internode length and 100-grain weight^57^, although stress conditions can negatively affect these traits. Early flowering characteristics, such as early-morning flowering (EMF), can mitigate heat-induced sterility and enhance yield under thermal stress^58,59^. Moreover, the Ef-cd locus has been shown to shorten maturity duration while improving nitrogen deficiency and photosynthetic performance^60^. These findings collectively underscore the complex interplay of genetic and environmental factors influencing rice yield and stress resilience^61^.

Salinity stress markedly reduced chlorophyll content, total soluble proteins, and total soluble sugars in rice plants (Fig. 6a-c), reflecting a disruption in photosynthetic efficiency and metabolic activity. However, application of Se-NPs and ZnO-NPs via fertigation effectively alleviated these detrimental effects by preserving chlorophyll content and enhancing photosynthetic performance of rice. This could be attributed to their role in stabilizing thylakoid membranes, preserving chloroplast structure, and protecting photosystem II (PSII) from salinity-induced photoinhibition. Previous studies have demonstrated that salinity-induced oxidative stress impairs protein synthesis, disrupts organelle integrity, alters metabolic pathways, and leads to the degradation of photosynthetic pigments, such as chlorophyll^62^. Chlorophyll a demonstrated a stronger tie to growth traits in comparison to chlorophyll b (Fig. 8), possibly due to its central role in light harvesting. Additionally, salinity stress damages the thylakoid membrane due to Na^+^ accumulation and causes structural enlargement of chloroplasts^63^, while also suppressing non-photochemical quenching, PSII quantum efficiency, and electron transport rate^64^ collectively reducing photosynthetic output. Our findings are consistent with the earlier reports on mitigating the effects of NPs under salinity stress in various crops, including *Helianthus annuus*^65^, *Moringa olifera L.*^66^, *Zea mays*^67^, *Triticum aestivum*^68^, and *Sorghum bicolor*^17^. Notably, Se-NPs and ZnO-NPs significantly enhanced the levels of soluble protein and total soluble sugar in salinity stressed plants, in agreement with prior findings. Zn plays a pivotal role in carbohydrates and starch metabolism, either directly or indirectly, or by modulating enzymes involved in starch to glucose or sugar conversion^69^. Soluble sugars, as small organic osmolytes, also known as osmoprotectants, that aid in maintaining cellular homeostasis under salinity stress^70^. They maintain cell turgor, protect cellular proteins, and stabilize membranes under osmotic stress, thereby enabling the plant to retain metabolic function during salinity.

Salinity stress adversely affects plants through osmotic imbalance, ion toxicity, and nutrient deficiency^71^. In response, plants adopt adaptive strategies their metabolic functions^72,73^. In our study, significantly elevated MDA level, which is an indicator of lipid peroxidation and oxidative damage, highlights increased membrane injury in stressed plants. However, Se-NPs and ZnO-NPs substantially reduced MDA accumulation, in agreement with previous findings in *Solanum lycopersum*^74^ and *Zea mays*^75^. Additionally, proline levels increased significantly under salt stress and were further enhanced by nanoparticle treatment^76^. Proline acts not only as an osmolyte but also plays a critical role in protein/enzyme and membrane stabilization, scavenging free radicals, and buffers cellular redox state^77,78^. Enhanced proline accumulation under NP treatment suggests improved osmotic adjustment and antioxidative defense, consistent with earlier studies^79–81^. Salt induced oxidative stress also triggered overproduction of ROS, including superoxide radical (O_2_^•−^), singlet oxygen (^1^O_2_), hydrogen peroxide (H_2_O_2_), and hydroxyl radical (OH^•^)^82,83^. Histochemical staining by DAB and NBT confirmed that nanoparticle-treated plants had significantly lower ROS accumulation compared to untreated stressed plants, indicating improved oxidative stress^84^.

**Fig. 10:**
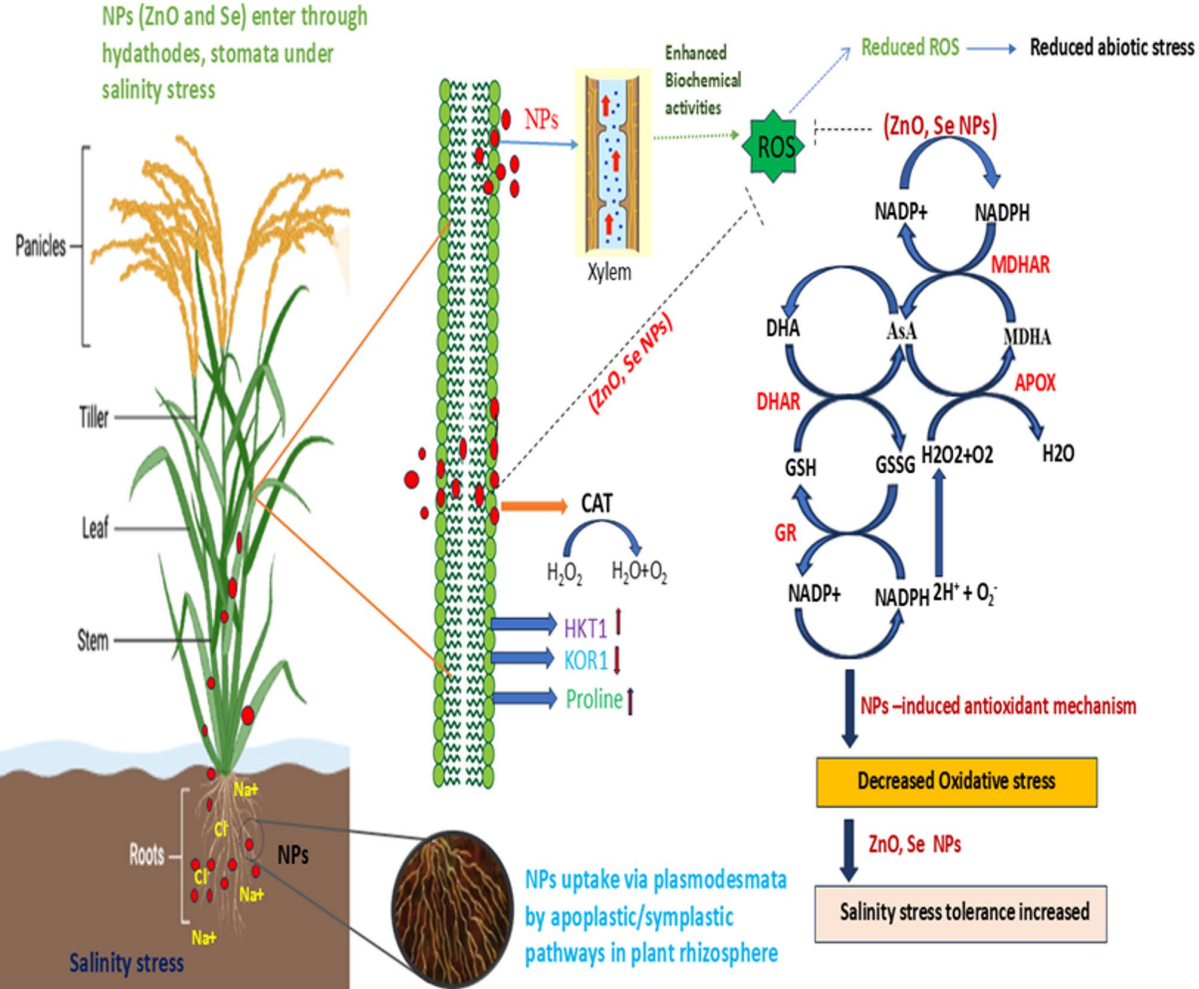
A conceptual illustration depicting the proposed mechanism by which nanoparticle (NP) application mitigates salinity stress in rice plants. Under saline conditions, excessive Na⁺ disrupts ion homeostasis and induces oxidative stress through the overproduction of reactive oxygen species (ROS) such as H₂O₂, O₂•⁻, and OH•. The application of Se-NPs and ZnO-NPs enhances the plant’s antioxidant defense system, which includes enzymatic antioxidants such as superoxide dismutase (SOD), catalase (CAT), ascorbate peroxidase (APX), and glutathione reductase (GR). Moreover, monodehydroascorbate reductase (MDHAR) and dehydroascorbate reductase (DHAR) play crucial roles in regenerating ascorbate (AsA) from its oxidized forms, maintaining the AsA–GSH (ascorbate–glutathione) cycle. This cycle is essential for scavenging ROS and restoring cellular redox balance. DHAR catalyzes the reduction of dehydroascorbate (DHA) to AsA using reduced glutathione (GSH) as an electron donor, while MDHAR reduces monodehydroascorbate (MDHA) back to AsA using NAD(P)H. Glutathione reductase (GR) maintains the GSH pool by reducing oxidized glutathione (GSSG) to GSH. The coordinated activity of these enzymes enhances the recycling of antioxidants, reduces lipid peroxidation (as indicated by decreased MDA levels), supports ion regulation (improved K⁺/Na⁺ ratio), and ultimately improves growth, stress tolerance, and grain mineral content in rice under salinity stress. HKTT-1, a Na^+^ transporter, is also upregulated, reducing sodium accumulation in shoots and maintaining ionic balance. KOR1 supports cell wall remodelling, preserving root growth and structural integrity under osmotic stress. Proline biosynthesis further contributes to osmotic adjustment, ROS scavenging, and cellular protection.

High salinity conditions are known to suppress antioxidant enzyme activities in crops, thereby impairing the plants ability to detoxify the ROS^85^. However, the application of green-synthesized NPs has been shown to enhance the antioxidant systems under stress, mitigating oxidative damage^15,86,87^. In the present study, salinity stress alone triggered increased activity of key antioxidant enzymes (SOD, POD, APX, and CAT) in rice plants, suggesting an intrinsic defensive response to elevated ROS levels. This is in agreement with Zhang et al.^88^ who reported enhanced activity of CAT and POD enzyme activity in salinity-stressed plants. Further treatment with Se-NPs and ZnO-NPs significantly an enhanced ROS-scavenging capacity. This suggests activation of ROS-scavenging pathways, likely mediated by enhanced expression of stress-responsive genes and upregulation of antioxidant defense signaling cascades. These findings align with previous studies where ZnO-NPs and Se-NPs improved antioxidant enzyme function in *Solanum melongena* and *Physalis alkekengi* L, respectively, under salinity stress^89,90^. Enzymes like CAT and POD play a crucial role in ROS detoxification, wherein CAT decomposes H_2_O_2_ into water and oxygen^91^ while POD further neutralizes H_2_O_2_ via electron donors^92^. The increased activity of these enzymes following NP application suggests enhanced redox homeostasis and stress resilience in rice plants. However, studies suggest NPs may alleviate salt stress by reducing oxidative damage, enhancing photosynthesis, regulating ion homeostasis, and influencing gene^93^ (Fig. 10). Salt stress disrupts the cellular ion balance by increasing Na^+^ and reducing essential ions like K^+^ and Ca^2+^, leading to oxidative stress^57^. Se-NPs and ZnO-NPs may help restore this balance by influencing ion transporter activity, enhancing selective ion uptake, and facilitating vacuolar sequestration of Na^+^. Although the in-depth molecular mechanisms remain unclear, evidence suggests that specific NPs can modulate stress-related gene expression, warranting further investigation^77^.

Furthermore, the application of ZnO-NPs has been associated with enhanced uptake of crucial micronutrients, such as Fe and Zn^52^, which is consistent with our results. The uptake of essential micronutrients such as Zn, Fe, P, and K in rice plants was notably influenced by salinity stress under ZnO-NPs treatments. This variation could be attributed to alterations in root architecture, root-soil interaction, and the efficiency of nutrient absorption and translocation^94^. Our study revealed a significant accumulation of minerals in the grains of both stressed and non-stressed plants treated with Se-NPs and ZnO-NPs. This supports the potential of NPs not only for stress mitigation but also for nutritional enhancement through biofortification. Notably, Se-enriched rice has been proposed as a useful approach to combat Se deficiency in the human population with the potential to supply 100–200 µg Se per day through regular dietary intake^95^. Therefore, biofortification of rice grains with Se and Zn via nanoparticle treatment holds promise for improving both the plant’s resilience and human health outcomes.

Significantly, the combination of Se-NPs and ZnO-NPs outperformed individual treatments in nearly all traits measured. This suggests a synergistic interaction where selenium enhances antioxidative capacity, while zinc supports structural and metabolic processes. Their co-application may also improve nanoparticle uptake efficiency, intracellular signalling, or complementary activation of stress-related pathways.

## Conclusion

The contemporary study focused on the influence of green-synthesized Se-NPs and ZnO-NPs on the growth, physiology, and yield of rice plants subjected to salinity stress. Our findings demonstrate that rice plants are adversely affected by salinity stress, impaired nutrient uptake, and a decrease in overall productivity. Salinity stress also induced oxidative stress, increasing H_2_O_2_, MDA, and antioxidant enzyme activity. Exogenous application of Se-NPs and ZnO-NPs, both individually and in combination, significantly mitigated the detrimental effects of salt stress. These NPs improved physiological functions, enhanced photosynthesis and nutrient accumulation, and promoted overall plant growth and yield of rice. Notably, our results demonstrate that the combined application (Se-NPs 10 mgL^− 1^ + ZnO-NPs 20 mgL^− 1^) was significantly more effective than the individual treatments in enhancing rice plant resilience under salinity stress. This improved efficacy can be attributed to the synergistic interaction between selenium and zinc, which not only improved the scavenging of ROS and the regulation of antioxidant enzyme activities but also contributed to better nutrient uptake, photosynthetic performance, and overall plant vigor. As a result, combined treatment led to markedly improved plant health, growth, and yield compared to either nanoparticle applied alone. Additionally, our results underscore the potential of green-synthesized NPs as effective nano-fertilizers for improving crop performance under salinity stress.

While the results underscore the potential of SE-NPs and ZnO-NPs as nano-fertilizers, long-term environmental impacts, such as nanoparticle accumulation in soil or food chains, remain a concern and warrant further investigation. Practically, these nanoparticles can be integrated into precision agriculture or sustainable nutrient management strategies, particularly in regions prone to salinity. Future research should include field-scale validation to confirm the real-world applicability of these findings, as well as molecular studies, such as gene expression and signaling pathways, to better understand the underlying mechanisms of nanoparticle-mediated stress mitigation. Additionally, evaluating their role in addressing other abiotic and biotic stresses will broaden their utility for climate-resilient agriculture.

## Data Availability

The datasets used and/or analyzed during the current study are available from the corresponding author on reasonable request.

## Abbreviations

APX: Ascorbic acid peroxidase
CAT: Catalase
Chl a: Chlorophyll a
Chl b: Chlorophyll b
DAB: 3,3-diaminobenzidine
DF: Days to flowering
FW: Fresh weight
GS: Grains per spike
GW: Grain weight
MDA: Malonaldehyde
NBT: Nitroblue tetrazolium chloride
NP: Nanoparticle
PH: Plant height
POD: Peroxidase
RL: Root length
ROS: Reactive oxygen species
Se-NP: Selenium nanoparticle
SL: Spike length
SOD: Superoxide dismutase
SP: Number of spikes per plant
TL: Number of tillers
Total Ch: Total chlorophyll
ZnO-NP: Zinc oxide Nanoparticle
